# Supplementary material for: Single-cell and spatially resolved interactomics of tooth-associated keratinocytes in periodontitis
Source: Nat Commun. 2024 Jun 14;15:5016. doi: 10.1038/s41467-024-49037-y (PMC11178863; doi:10.1038/s41467-024-49037-y)
Supplement: Supplementary file 1 — Supplementary Information [file 41467_2024_49037_MOESM1_ESM.pdf]

Supplementary Figure 1

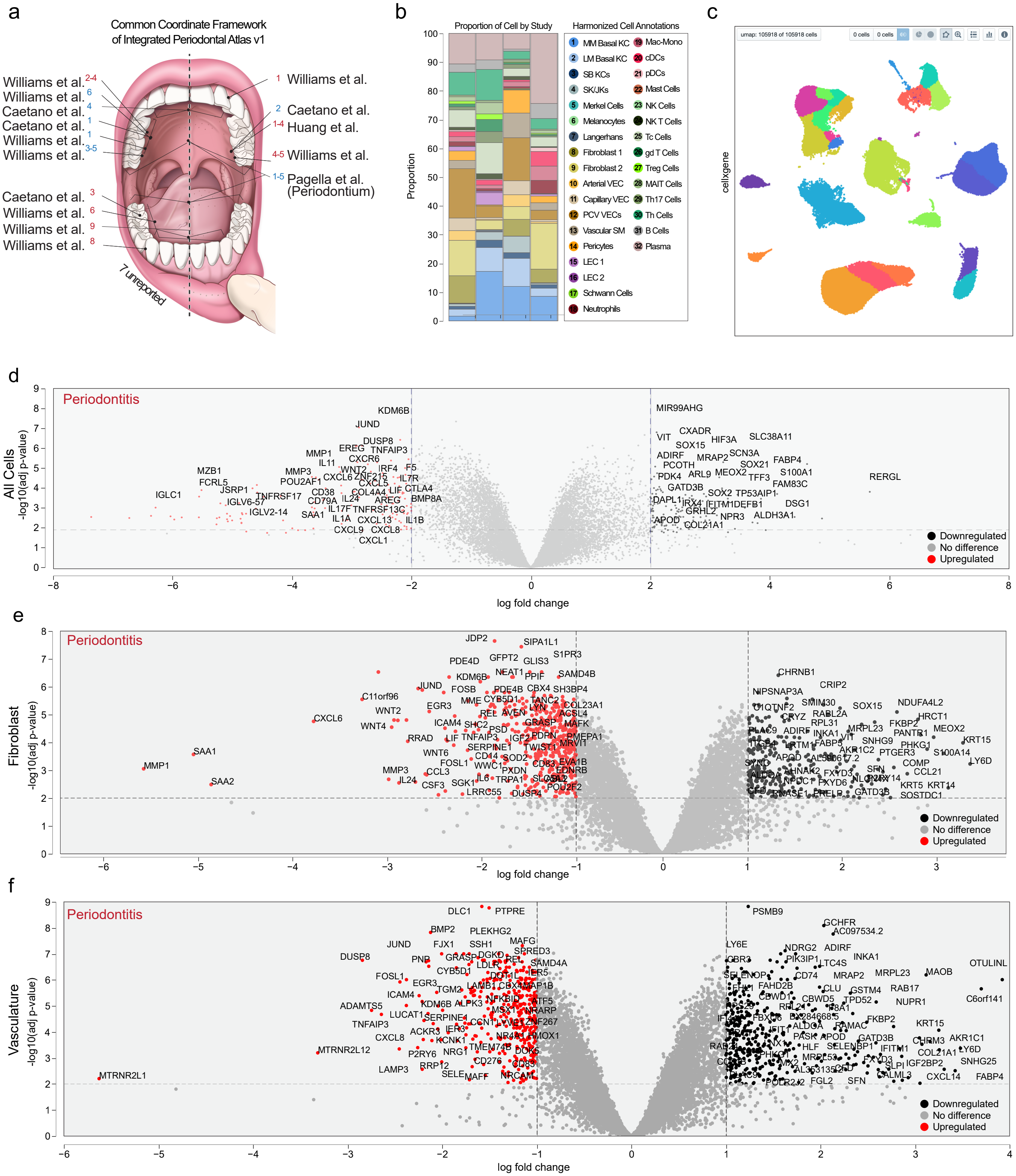

Supplementary Figure 1 | Metadata and differentially expressed gene analyses of the integrated periodontitis atlas. (a) Common coordinate framework annotations were included from each study sample here visually and in Supplementary Table 1, including descriptions of regions (anterior versus posterior), tooth type (incisors, canines, premolars, or molars), and specific tooth number (universal numbering system) when available. (b) Cell type (Tier 3 annotation) proportions for each of the four studies. (c) For this study, Cellenics®, which is an open-source tool for single-cell RNA sequencing analyses (https://github.com/hms-dbmi-cellenics), was used for integration, data analysis, and some plot generation. For public use, Cellenics® and CELLxGENE were linked, conserving the UMAP coordinates in the CELLxGENE space. Metadata from Supplementary Table 1 was incorporated into CELLxGENE (https://cellxgene.cziscience.com/) to further enhance public utility. (**d-f**) Pseudobulk analysis of differentially expressed genes (DEGs) in periodontitis using all (d) and Tier 1 cell annotations of (e) fibroblasts and (f) vasculature are shown via volcano plots. Only some DEGs are highlighted; the full list is in Supplementary Table 1. Abbreviations: see Figure 1 legend.

Supplementary Figure 2

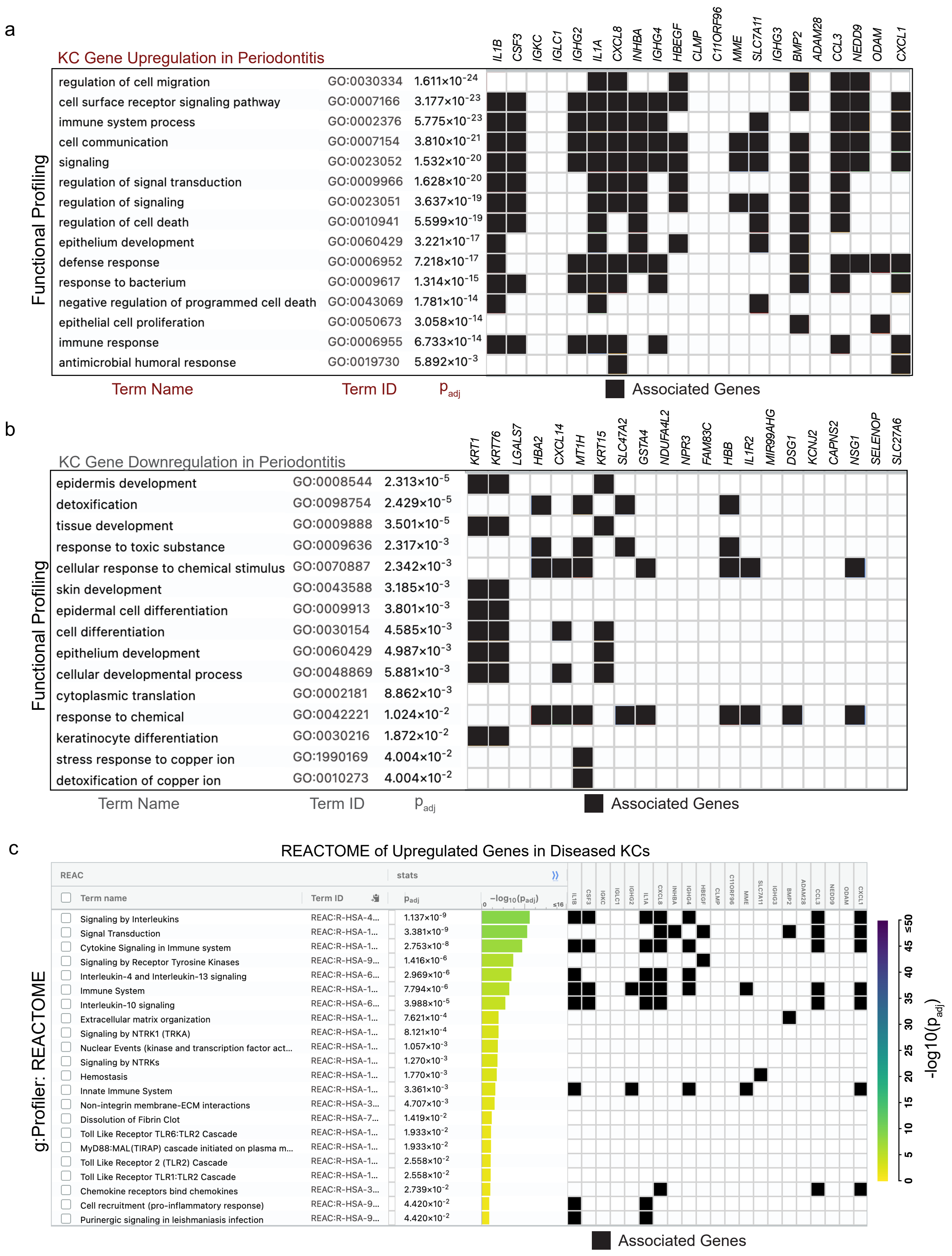

Supplementary Figure 2 | Keratinocytes are predicted to play important roles in the host response to periodontitis. (**a-b**) (a) Assessing biological pathways using g:Profiler, key processes that are upregulated in disease include cell migration, cell signaling, cell death, and cell responses to bacteria. (b). Key processes that are downregulated include tissue differentiation and development, protein translation, and stress responses. This is just an example of the g:Profiler data and key genes attributed to these pathways; however, these data suggest an active immune signaling role for keratinocytes in periodontitis beyond simply wound healing. (c), The reactome of keratinocytes in periodontitis further emphasizes their active role via cytokine signaling, immunoregulation, and immune cell recruitment which was observed as presented in Figure 2. Abbreviations: see Figure 1 legend.

Supplementary Figure 3

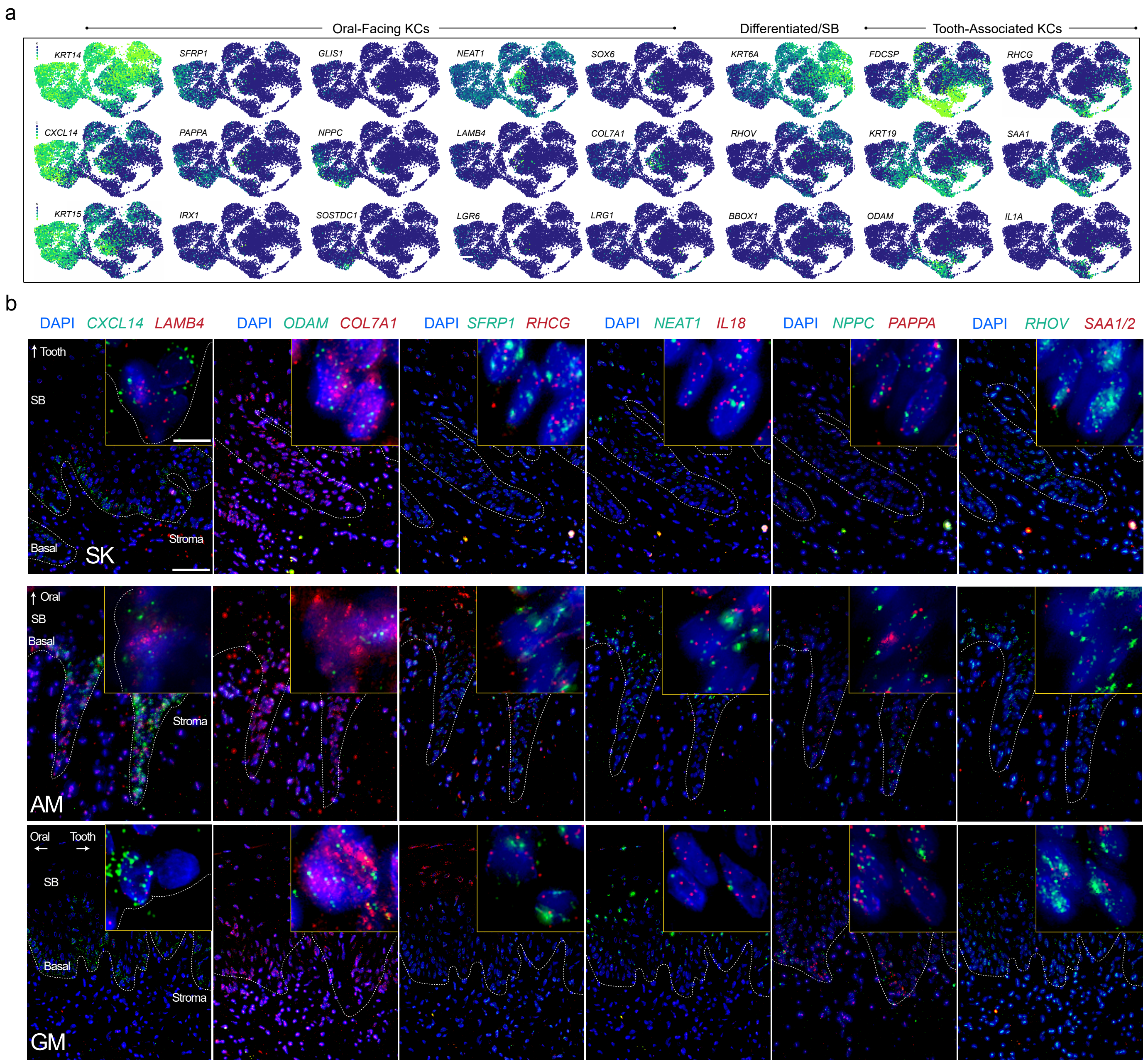

Supplementary Figure 3 | Human gingival keratinocytes contain distinct subpopulations. (a) Using UMAPs to demonstrate cell marker enrichment, keratinocytes are defined by *KRT14* expression. JK and SKs are defined by *KRT19, FDCSP, RHCG, SAA1/2, IL1A,* and *ODAM.* (b) Validation of markers using a custom 12-plex ISH panel in non-keratinized oral mucosa (alveolar mucosal keratinocytes, AM) and non-keratinized oral mucosa near the tooth surface (gingival margin, GM; sulcular keratinocytes, SK). Scale bars: (b) 25 µm. Sequential sections from samples were used (n = 3 health).

Supplementary Figure 4

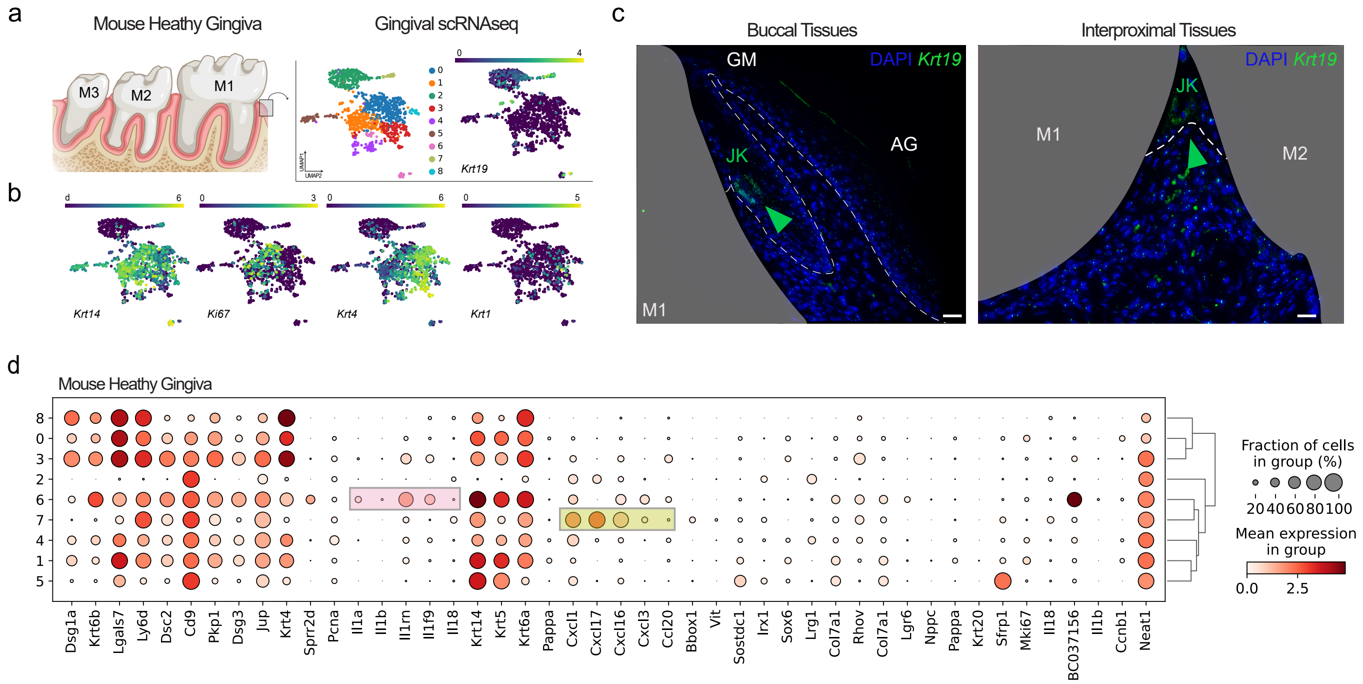

Supplementary Figure 4 | Mouse gingival keratinocytes also contain distinct subpopulations but their similarity to human cell types is less clear. (a) Single-cell RNA sequencing (scRNAseq) was performed on adult healthy gingival tissues from mice; the UMAP represents the subclustering of murine keratinocytes. (b) Using Louvain clustering, *Krt14*$^{+}$/*Krt19*$^{+}$ cells were found in small proportion to differentiated keratinized mucosal cells. (c) Though *Krt19* was expressed as mRNA, Krt19 was not detected at the protein level. (d) Looking at similar cell signatures discovered in humans, many markers are not specific, though there appears to be some expression of immune markers in Krt19 (Cluster 7, yellow box), including *Cxcl1, Cxcl16, Cxcl17,* and *Cxcl3.* Another cluster (6, pink box) expressed some interleukin inflammatory markers unique to cluster 7 that are shared in humans. Abbreviations: Molar (M); see Figure 1 legend. Scale bars: (b) 50 μm, (c) 100 μm. Illustration from (a) created with BioRender.com.

Supplementary Figure 5

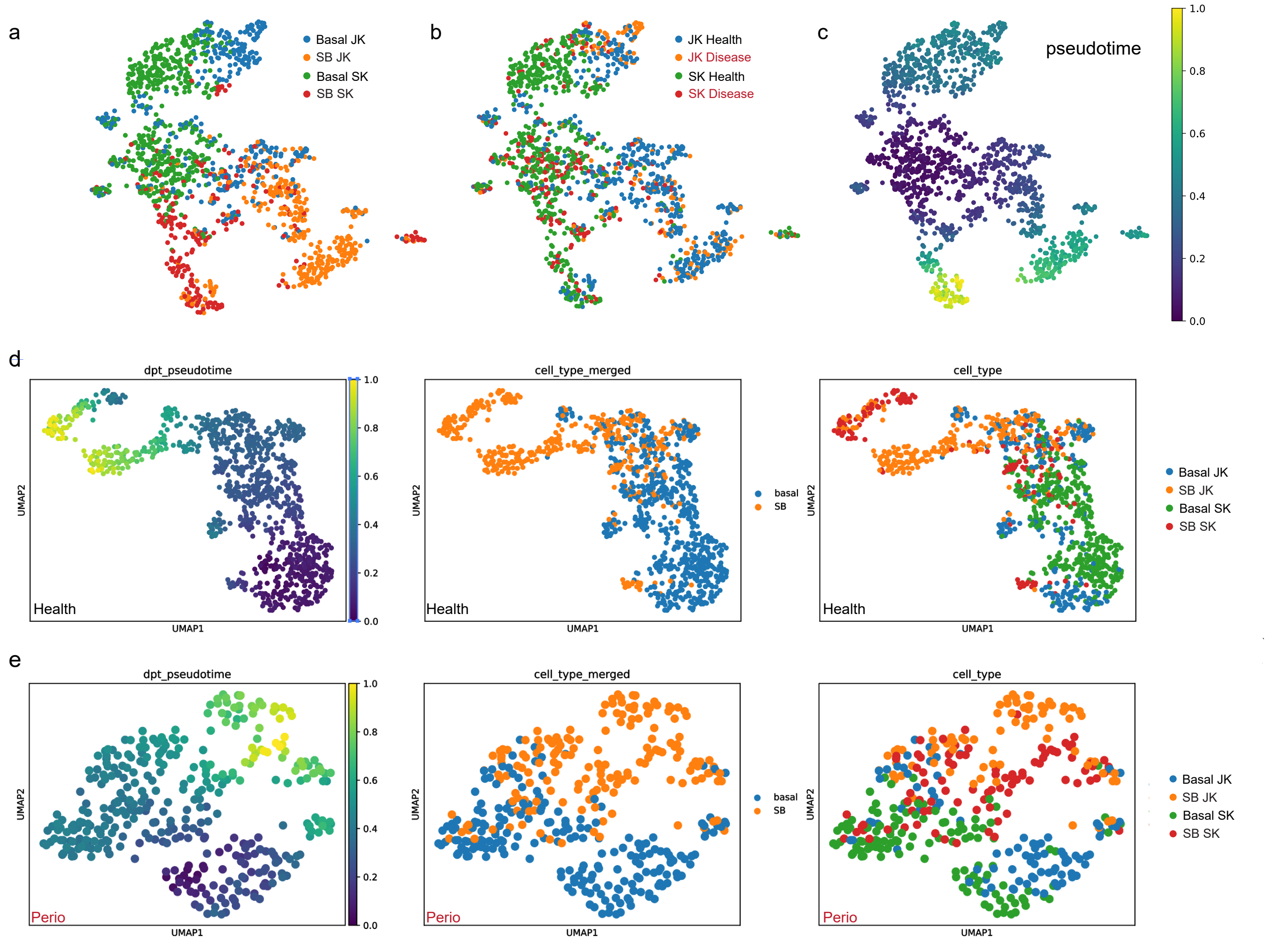

Supplementary Figure 5 | PAGA analysis of *KRT19*-high keratinocytes shows trajectory analysis towards terminally differentiated cells. (**a-c**) KRT19-high JK, SK, and GM keratinocytes (KCs) were subclustered for further annotation using PAGA. (**d-e**) Expanded PAGA plots for health (d) and periodontitis (e). Trajectory analysis of (d) and (e) showed that terminally differentiated SK/JK SB cells were present in health and periodontitis.

Supplementary Figure 6

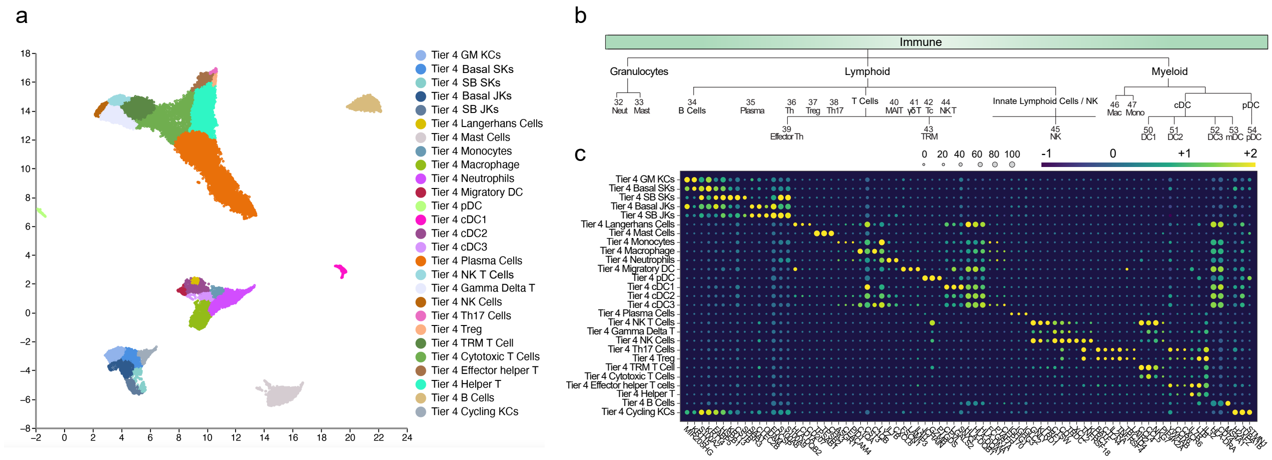

Supplementary Figure 6 | CellChat reveals up- and downregulated communication between cell subpopulations in periodontitis. (**a-c**) (a) Using CellTypist, we created a Tier 4 UMAP to reflect immune cell types of the periodontium. We further refined the first draft annotation of innate and adaptive immune cell subpopulations (b) that were included in the CellChat analysis[75] from Figure 3. (c) Markers are included for each cell subpopulation. Abbreviations: Tissue Resident Memory T Cell (TRM T); also see Figure 1 legend.

Supplementary Figure 7

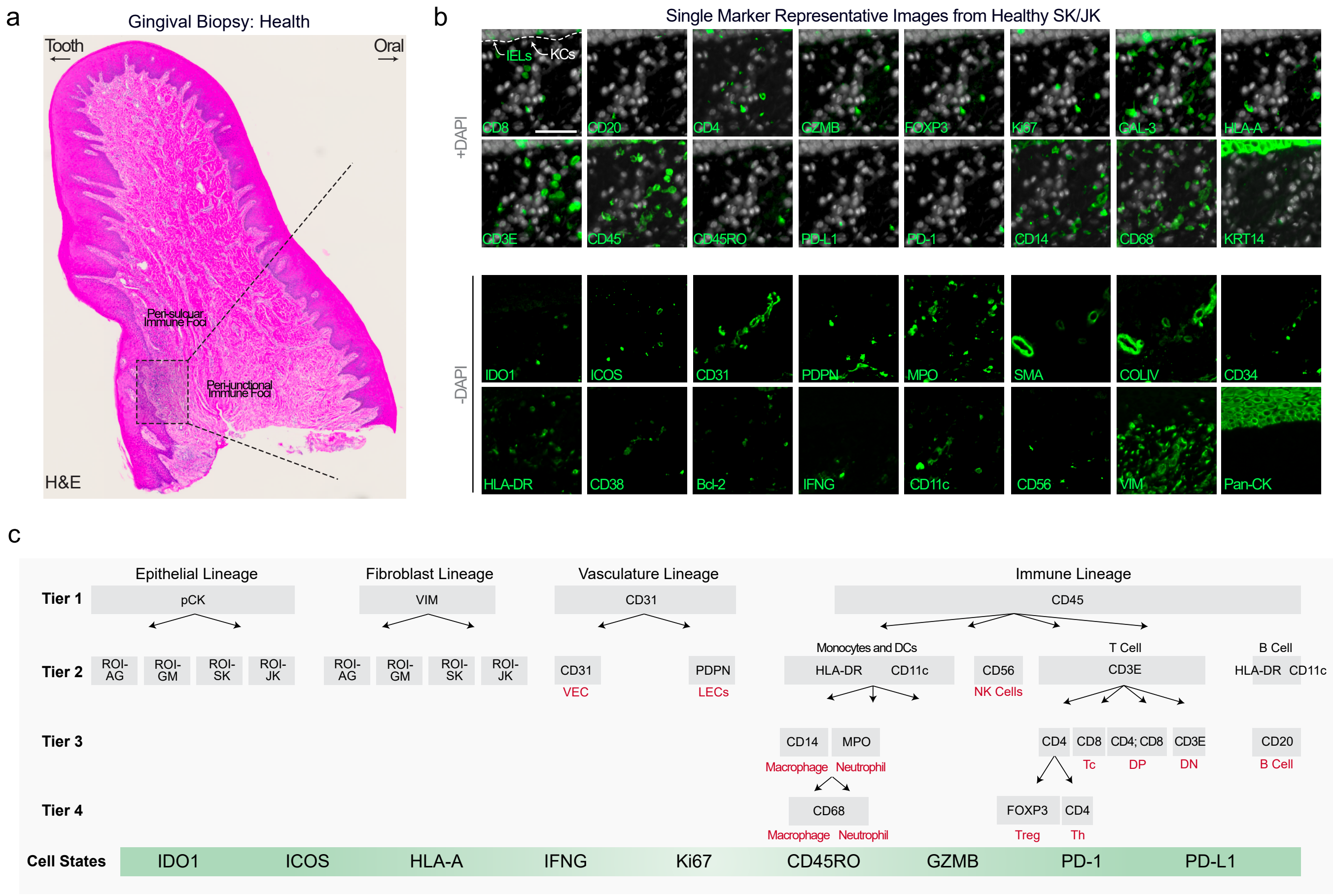

Supplementary Figure 7 | Validation and methods for analysis of oral spatial proteomics datasets. (a) Orientation of health tissue with tooth and oral facing sides allows for peri-junctional immune foci characterization also in health. (b) Validation of single markers in the health periodontal niche. As expected, there are few adaptive immune cells and minimal exhaustion phenotypes expressed in healthy tissues, though the peri-junctional space does express some of those identities and states in health. (c) Tier assignment algorithm for multiparameter cell type assignment and cell state analysis used in Figure 2. All cell states except Ki67 (cycling) relate to immune activation. Abbreviations: see Figure 7 legend. Scale bars: (a) 250 μm; (b) 50 μm.
