# Supplementary material for: Single-cell and spatially resolved interactomics of tooth-associated keratinocytes in periodontitis
Source: Nat Commun. 2024 Jun 14;15:5016. doi: 10.1038/s41467-024-49037-y (PMC11178863; doi:10.1038/s41467-024-49037-y)
Supplement: Supplementary file 3 — Description of Additional Supplementary Files [file 41467_2024_49037_MOESM3_ESM.pdf]

Description of Additional Supplementary Files

File Name: Supplementary Data 1
Description:
Clinical metadata, tiered marker genes, gene expression change, and mycoplasma testing of primary gingival keratinocytes.

File Name: Supplementary Data 2
Description: Cellphone DB, CellChat, and bacterial enrichment data.
